# Midwives’ experiences of an organizational change in early postpartum care services in Norway: A qualitative study

**DOI:** 10.18332/ejm/147746

**Published:** 2022-04-19

**Authors:** Trude Levorstad, May-Sissel Saue, Anne Britt V. Nilsen, Eline S. Vik

**Affiliations:** 1 Department of Health and Caring Sciences, Faculty of Health and Social Sciences, Western Norway University of Applied Sciences, Bergen, Norway

**Keywords:** postpartum period, midwifery, organizational changes, maternity care, home visits

## Abstract

**INTRODUCTION:**

The length of postpartum hospital stays is decreasing internationally. The ongoing organizational change from hospital to home-based postpartum care implies the promotion of family-centered care for healthy women, their babies and families. The aim of the study was to explore midwives’ experiences of an organizational change in early postpartum care services from hospital to home-based care in Norway.

**METHODS:**

Ten midwives participated in two separate focus-group interviews. Systematic Text Condensation (STC) was the method used to analyze the data.

**RESULTS:**

The midwives in the current study described that the organizational change in early postpartum care services from hospital to home-based care was characterized by: 1) unclear leadership, 2) increased continuity of care and professional growth, and 3) the midwives being solution-oriented.

**CONCLUSIONS:**

The midwives had both positive and negative experiences of an organizational change in early postpartum care services from hospital to home-based care in Norway. The study indicates a need for increased resources, training in new tasks and employees being more included when changes in midwifery practice are planned and implemented. The current study contributes knowledge of relevance to those planning to start home-based postpartum care, which may contribute to improving quality of care, employees’ satisfaction and prevent burnout.

## INTRODUCTION

The length of postpartum hospital stays is decreasing internationally^1^. The ongoing organizational change from hospital to home-based postpartum care implies the promotion of family-centered care for healthy women, their babies and families^2,3^. There is ongoing controversy among healthcare providers and policy makers whether shorter postpartum hospital stays are harmful or beneficial to maternal, paternal and newborn health^1,4,5^. There is no clear evidence of the best time to transfer the care of women and their babies from hospital care to community care after birth and the definition of early discharge varies internationally^1^. A recent Cochrane review highlights that early discharge is associated with an increased risk of babies being readmitted to hospital, however, the risk of severe uncommon outcomes such as maternal and neonatal death following early discharge remains uncertain^1^. Both international^2^ and national guidelines^3^ recommend that hospital discharge be contingent upon the health and wellbeing of both mother and baby, the women’s preferences, as well as the level of support available after discharge. Today, healthy women and their babies are commonly discharged four to twenty-four hours after birth followed by a home visit within the first days of returning home^1,3^. Consequently, the responsibility of community midwives has changed from their not being formally involved in postpartum care to their being the primary caregivers for healthy mothers and babies in Norway and in other middle- and high-income countries^1,3^.

The postpartum period covers a critical transitional period for a new family^1^. Especially in the immediate postpartum period (i.e. first two weeks postpartum), both mother and baby have a range of needs that require attendance, including follow-up on breastfeeding, newborn weight loss and jaundice, and maternal physical and psychological wellbeing^1^. New parents highlight their own sense of security as a crucial aspect of quality care^6,7^ and midwives want a leader who advocates for midwifery^8^.

Little research has been conducted into midwives’ experience of organizational change in postpartum practice. It is important to learn from the participants’ experience of organizational change processes so that future organizational changes can allow others to benefit. Therefore, we set up the study with the aim of exploring midwives’ experience of an organizational change in early postpartum care services from hospital to home-based care in Norway.

## METHODS

This is a qualitative study. The RATS Checklist (Relevance, Appropriateness, Transparency, and Soundness) was used for strengthening the reporting of the data^9^.

### Setting

The midwives in this study were employed by municipal healthcare centers in one of Norway’s largest cities. In 2017, the municipality applied for grant funding from the Norwegian Directorate of Health with the intention of implementing home-based postpartum care in their services. The financial resources were transferred the following year (2018), and shortly after the centers went from offering solely antenatal care to also offering home-based postpartum care by midwives to families with a newborn baby. Initially, the organizational change in early postpartum care services from hospital to home-based care was led by a midwife, however, the midwife was only employed in a small part-time position. The organizational change involved the introduction of postpartum home visits by midwives, which took place within 3 to 6 days after birth. Most families who were offered home-based care after early discharge from the hospital had a healthy pregnancy and delivered their baby vaginally at term. In 2018, women who gave birth vaginally in the area where the study was conducted, were on average discharged 2.1 days postpartum^10^. Not all women in the municipality were offered home-based postpartum care despite an early discharge from hospital. However, some women were prioritized, such as first-time mothers, women with a migrant background and women who had not had follow-up by a midwife during pregnancy. At the time of the interviews, a standard home visit had a limit of two hours, including establishing contact with the families by telephone, preparing the visit by reading medical records, traveling time and documentation after the home visit. The visits were documented in both the mother’s and the baby’s medical journals. After the initial 7–10 days postpartum, the role as main caregiver was transferred to a public healthcare nurse in the municipality. Today, community midwives offer home visits to new families regardless of who provided antenatal care.

### Participants

All midwives who were employed in the municipally where the organizational change was implemented were invited to join the study. The final sample included 10 midwives, which represents almost half of all midwives eligible for the study. The midwives’ background characteristics can be seen in Table 1. In addition to their work experience from municipal healthcare centers, the midwives had experience of maternity wards, hospital-based postpartum wards, outpatient clinics, medical facilities and private practice, including home birth services. Furthermore, some of the midwives had a formal specialization in a variety of areas such as breastfeeding and public health nursing.

**Table 1:** Background characteristics of study participants (N=10)

*Characteristics*	*Median (range)*
Percent (%) employment in current position at municipal healthcare center	80 (40–100)
Seniority as midwife (years)	26.0 (1.5–31.0)
Number of pregnant women cared for at the municipal healthcare center (by given midwife per month)	13 (7–16)

### Data collection

The focus-group interviews were conducted in the autumn of 2019, one year after the organizational change. Seven midwives participated in the first focus-group interview, and three midwives participated in the second focus-group interview. Both interviews were conducted at the Western Norway University of Applied Sciences. A semi-structured interview guide was prepared which contained 10 open-ended questions (Table 2). The open-ended questions were chosen after a discussion among the authors. The Yukl theory on organizational change^11^, in light of which the results are discussed, was chosen after the interviews were conducted. The first and second authors conducted the interviews; they moderated one interview each, during which the other author functioned as secretary, taking notes and asking follow-up questions as appropriate. The last author followed the interviews as an observer. The interviews lasted about 50 and 80 minutes, respectively. Both interviews were recorded on tape. The atmosphere during the interviews can be described as engaging. Focus-group interviews were chosen to enhance and capture rich discussions among the participants^12^.

**Table 2:** Interview guide

**Question 1**	Can you explain how you experience going on home visits after birth?
**Question 2**	How has the organizational change process been in practice? How has it been for you to change from the practice you had before to being able to offer home visits for new families after birth?
**Question 3**	What benefits have you experienced with the home visits?
**Question 4**	What challenges have you experienced with the home visits?
**Question 5**	Explain what you consider to be the most important work tasks when on home visits after birth.
**Question 6**	How often do you visit women you have cared for during their pregnancy? What are your reflections on this?
**Question 7**	How do you experience the collaboration with public health nurses and general practitioners in the municipality/maternity wards in hospitals?
**Question 8**	Do you find that there are any advantages and/or challenges in terms of travel/transport related to home visits? Do you use your own car/company car or spend money on travel, how much time do you spend, etc.
**Question 9**	What advice do you have for others who plan to start with home visits after birth?
**Question 10**	Is there anything else related to the topic that you feel is important to address? Please explain.

### Analysis of the data

The interviews were transcribed verbatim by the first and second authors. Systematic Text Condensation (STC) was the method used to analyze the data^13^. STC is a pragmatic method for thematic cross-sectional analysis of qualitative data, principally to develop knowledge of participants’ lived experience of a given phenomenon^14^. STC was carried out in the following four steps. In the first step, we read through the interviews in a ‘bird's eye view’ to form an overall impression of the data material. In line with the method^14^, we identified seven preliminary themes as follows: 1) positive for maternity care, 2) difficult start-up, 3) midwives feel alone, 4) time-challenges, 5) midwives are flexible, 6) midwives have broad competence, and 7) cooperation challenges. In the second step, we identified meaning units describing the midwives’ experience of the organizational change in early postpartum care services from hospital to home-based care and sorted them into three different code groups. In the third step, we abstracted the content of the three code groups of meaning units and formed subgroups describing different aspects of the content of the code groups. In the fourth step, we used the text condensates from step three as the basis for our final analytic text. Direct quotations were used to show key messages from each subgroup. After assessing the data independently, the first and second authors reached consensus before including the third and fourth authors in the discussion. All authors took part in all steps of the analysis and an overview of the three last steps (2–4) of the analysis can be seen in Table 3.

**Table 3:** A short presentation of steps 2, 3 and 4 of the Systematic Text Condensation (STC) process

**Step 2. Meaning units were sorted into code groups**		
1. The midwives were positive about starting with home-based postpartum care, as the change in practice provided continuity in their work and the opportunity to use more of their competence as midwives.	2. The midwives experienced practical challenges in the start-up phase, called for a midwife leader and found themselves with little opportunity to influence the organizational change process.	3. The midwives experienced practical challenges but were solution-oriented in collaboration with other health professionals.
**Step 3. The content in each of the three code groups was sorted into subgroups**		
1. Continuity of care 2. Self-development and variety in the work and being a source of security 3. Number of families you visit at home	1. Difficult start-up 2. Lack of involvement, feeling alone and lack of midwife leadership 3. Many tasks, little time 4. Advice for others who are going to start with home visits and hopes for the future	1. Time resources 2. Challenges with transport to and from the home visits 3. Flexible and solution-oriented in their work with colleagues
**Step 4. Results**		
The names of the code groups were retained as headings in the final analytic text. In the results, each subgroup is represented by a paragraph of analytic text illustrated by a direct quotation to indicate a key message from the subgroup.		

### Reflexivity

All authors in the current study are midwives. Notably, we find several of the challenges described by the midwives in our study recognizable from practice. While we, as researchers, have aimed for open-mindedness, our own experiences as student midwives and midwives may have influenced our research. At the time of the interviews, the first and second authors were Master’s degree students and the third and fourth authors were their supervisors.

### Ethical considerations

The study was approved by the Norwegian Centre for Research Data (NSD: Ref-714749) and the Regional Committees for Medical and Health Research Ethics (REK: Ref - 25044 REK West). The study was conducted according to the ethical principles outlined in the Helsinki Declaration^15^. The participants received written information about the study and signed a consent form before the focus-group interviews. Data collected were stored and handled on a secure research server using two-factor authentication, hosted by the Western Norway University of Applied Sciences. To protect the identity of the participants, the names used in this study are fictional.

## RESULTS

In this study, we identified the following three main themes: 1) Unclear leadership, 2) Increased continuity of care and professional growth, and 3) The midwives being solution-oriented.

### Unclear leadership

In the start-up phase, the midwives called for a midwife leader with more available time for the project and found themselves with little opportunity to influence the organizational change process. The midwives explained that they found the start-up of the organizational change difficult although they had known about the planned organizational changes for some time before the changes were set out in practice. When the date for the start-up arrived, the midwives explained how they immediately had to start going on postpartum home visits without being offered training, given more recourses, or included in the planning of the changes. According to the midwives, the rushed change in practice resulted in the midwives starting to offer postpartum home visits before they had been trained to do so. The midwives explained that training and clinical practice at the local hospital maternity ward were offered at a later stage. Midwives who had extensive experience as midwives said they lacked competence in postpartum care as a result of working in antenatal care for years. One of the midwives described the initial phase as follows:

*‘In our municipality, we had been wanting to start offering postpartum home visits for a long time. However, when it first happened, it happened very quickly. So, we were really thrown into it. We were simply told to just set aside time in our time schedules.’* (Petra)

The midwives talked about feelings of loneliness during the start-up phase and, in many ways, they felt as if they were self-employed. The midwives said that they had worked hard for the organizational change to be successful and they were passionate about their work. The midwives missed having a leader advocating for midwifery, who could have guided them professionally through the change process. They described feeling frustration at the lack of midwifery-oriented leadership, and said that the management apparently did not have a complete overview of midwifery tasks relevant to home-based postpartum care. A midwife with substantial experience of the municipal health service expressed it like this:

*‘We want a professional midwife leader who can argue on behalf of the midwifery service in the future and who will have more insight into how we can use our midwife resources. It is so important.’* (Kari)

The midwives provided advice for others who plan to start home-based postpartum care, and most of them said that good organization and careful planning before and during the implementation would be a good place to start the process. According to the midwives, the most important issue to address before starting home-based postpartum care is including the midwives at an early phase of the start-up. In addition, the midwives saw a need for up-to-date training for all midwives who change their practice. The midwives also discussed the importance of having a leader who can handle both the organization of the new service and guide the staff in their priorities. One midwife offered these suggestions:

*‘You need professional training related to home visits before you start, and leaders that can assist with both professional guidance and organization of the services, assessments and priorities.’* (Fride)

### Increased continuity of care and professional growth

The midwives were positive about starting with home-based postpartum care, as the change in practice provided continuity in their work and the opportunity to use more of their competence as midwives. The midwives talked about how caring for new families after birth created security for both themselves and the new parents, especially when they already knew each other from antenatal care. The new sense of continuity in care allowed the midwives to thrive professionally and obtain closure after following the women through their pregnancies. One midwife with extensive experience expressed it as follows:

*‘I find that the home visits lead to continuity in care, and many of those I visit I had cared for during their pregnancy. It makes it easier to understand and familiarize yourself with the woman’s experience and be able to help in a good way.’* (Sandra)

The midwives said that their expertise now covered more areas of midwifery than before they had started with home-based postpartum care. The organizational change had led to the midwives increasing their knowledge of breastfeeding and postpartum care. The midwives went on to discuss how their competence was continually developing. Being given the opportunity to perform home-based postpartum care led to positive variation in the midwives’ everyday work. They also found that the parents felt reassured knowing that their midwife from antenatal care would attempt to come on a home visit after birth. The midwives talked about how challenges identified during pregnancy were easier to handle when the new parents already knew the midwife. The start-up of home-based postpartum care was described as follows:

*‘Postpartum home visits are great for the women, and great for us too. So, it was about time we started with home visits, so to speak. I find that starting postpartum home visits has given me the opportunity to use my expertise in a wider range of midwifery care, partly because I have become much better at seeing the greater picture of what the women need, and thus it has also made me feel secure as a midwife. I think it is very satisfying to have some variation in work tasks; otherwise, you can easily feel burnt out.’* (Yvonne)

The midwives explained that it was more important to them to offer home-based postpartum care to a range of new families rather than seek out women they already knew. To ensure equitable postpartum care, the midwives preferred to refer to the families as ‘ours’ rather than ‘mine’. However, when possible, the midwives would attempt to care for women they already knew. In cases of special needs, the midwives would do what was in their power to ensure that a midwife already known to the woman in question went on a postpartum visit. One midwife explained the practice as follows:

*‘The most important thing is getting to go on postpartum home visits to as many new families as possible, without focusing too much on who cared for the woman during pregnancy. Of course, it is best if you can visit a woman who you cared for during her pregnancy. But, to keep the numbers up, we cannot allow ourselves to choose which women to visit.’* (Linda)

The midwives said that they always felt welcome when they went on postpartum home visits. A common opinion among the midwives was that new families in general are positive about welcoming professionals in their homes. Offering home-based postpartum care was seen as a great advantage for the family, and the midwives assumed the home environment made it easier for families to ask questions related to subjects such as breastfeeding and woman’s health. One midwife said that it was important to focus on the birth experience while not losing sight of the big picture of becoming a new family. She described it like this:

*‘I find that women often need to talk about their birthing experience. Even in cases of uncomplicated birth.’* (Heidi)

### The midwives being solution-oriented

The midwives experienced practical challenges but were solution-oriented in collaboration with other health professionals. The midwives saw limited time resources as a challenge in relation to home-based postpartum care. Furthermore, the midwives said it was challenging when they had to depart from their strict time schedule due to women needing extra care, such as women who had challenges with breastfeeding, a traumatic birth experience or signs of infection. One midwife described challenges related to a strict time schedule as follows:

*‘Having limited time and resources when you meet a woman at her most vulnerable and her tears are pouring down… It is very difficult. Such situations break my heart.’* (Sara)

The midwives said that transport to and from home visits was often a challenge. Means of transport had not been discussed with the midwives, thus each midwife had to find her own solution to such practical issues. Some midwives said that they were offered the use of an electric bicycle, but none of the midwives was offered the use of a car. Electric bicycles were often used with great success; however, the bicycles were less convenient for long distances, in winter or on rainy days. The midwives spent an excessive amount of time waiting for buses and taxis. Therefore, the midwives often used their own car. They said that the preparation of travel invoices was time-consuming, and that it was challenging to find and pay for parking, especially in the inner-city area. One midwife described the challenges with transport as follows:

*‘There are many challenges related to transport around the city. Are you going to walk, run or cycle, or maybe you should get a requisition to use a taxi? We have not received any information or assistance related to how we should get from one end of the city to the other.’* (Lisa)

The midwives went to great lengths to provide the new families with high-quality postpartum care. They solved problems as they arose and said that they aimed to be flexible and solution-oriented. If a midwife had questions related to the organization of home visits, the midwife would call other midwives in the municipality to discuss this, so that they could learn from one another’s experience.

Postpartum care was described as being a busy time for new families; after an early discharge from the hospital, families were expected to go back to specialist healthcare services for follow-up, including neonatal blood and hearing tests, and breastfeeding support. The midwives therefore focused on limiting further stress on the new families and suggested a further strengthening of the home-based postpartum care service. Such strengthening of the service could be accomplished by including additional tasks, such as offering the necessary blood tests, hearing tests and breastfeeding support at home. In the beginning, there were some challenges in obtaining medical records from the specialist health services, which complicated the process of planning individual care. However, the midwives said that communication improved after feedback was given to the specialist health services. The midwives pointed out that the specialist health services and the public health nurses were cooperative and helpful when contacted for advice and guidance. One midwife described the collaboration with the public health nurses as follows:

*‘It is very easy to communicate with the public health nurses. So, our cooperation is very good. If we are to say something about the challenges as well, then I would like to say that it can be a struggle to get the public health nurses to read the mother's medical journal [in addition to the baby's journal].’* (Lise)

## DISCUSSION

The midwives in the current study were overall positive towards starting with home-based postpartum care. During the start-up phase, the midwives experienced a lack of information from their leaders and opportunity for involvement in the organizational change processes. After the organizational change, the midwives practiced more of their competences as midwives and experienced greater continuity in their work. When new challenges arose during the organizational change in practice, the midwives discussed these among themselves and had a low threshold for asking each other for advice. An organizational change process will have a great impact at all levels of an organization, and the results from this study are therefore discussed in the light of the Yukl theory on organizational change^11^.

In the current study, start-up began without the midwives being sufficiently involved in the change process and the need for a leader advocating for midwifery was articulated. Similar to the situation described in the current study, the need for change is often decided and driven by top management^11^, and resistance to changes is one of several ways that employees may react to change processes in an organisation^16^. According to Norwegian law, an employer must ensure that all employees receive the training, practice, and instruction they need to carry out work in a safe manner^17^. Similar to our findings, a Norwegian study indicated that midwives want a leader who advocates for midwifery^8^. According to Yukl, involvement in decision-making contributes to greater engagement and increases the likelihood of success^11^. The Yukl theory is supported by an Australian study where they found that opportunities to influence decisions and a sense of support from leaders are factors conducive to midwives remaining in the profession^18^. To limit unwarranted variation in practice, the findings in our study suggest a change of postpartum practice should include a leader advocating for midwifery, an offer of relevant resources and a greater inclusion of employees in planning the change.

In line with the Yukl theory of organizational change^11^, the midwives in the current study explained that the experience of continuity, self-development and coping all contributed to their positive attitude towards home-based postpartum care. Our findings are supported by studies exploring midwives’ experience of postpartum care, which show that midwives are satisfied with the provision of postpartum care when their work involves the provision of person-centered care and continuity of care^19,20^. Midwives who experience continuity in their work have been found to report lower levels of burnout, depression and anxiety, and higher levels of professional identity and autonomy^21^. Our findings are also supported by a recent study from Norway which found that childbearing women emphasize continuity of care and safety as important aspects of maternity care^7^ and an Australian study identified close relationships with clients as a factor associated with midwives’ well-being^18^. A strong relationship between the midwife and the women may give rise to an experience of coping and safety for everyone involved^22-25^. Thus, the increased continuity and professional growth described by the midwives in our study may encourage women’s confidence in their own coping resources, and home visits by a midwife may provide an opportunity for women and midwives to conclude their relationship in a natural way, with feelings of closure for both.

While many of the midwives in our study had worked in antenatal care for years, it had been years since they had worked in postpartum care. A study from Sweden had similar findings, where midwives expressed frustration over no longer being the expert due to new guidelines and work tasks^20^. According to Yukl^11^, changes imply new ways of working, prompting a need for facilitation and the support of health professionals in order to stay abreast of evidence-based practices. Worldwide, midwives are encouraged to follow evidence-based practices^2,26^, which implies that one must accept that practice changes as guidelines are developed and updated. According to Yukl, resistance from employees should be regarded as an energy source in favor of the organizational change^11^. This is particularly relevant, as in our study we can look at the solution-oriented midwives’ efforts and commitment to the project as a positive contribution to the change process.

### Strengths and limitations

In qualitative studies, it is the content of the data collection and the processes during the interviews that underpin the study, not the number of participants^27^. A challenge of focus-group interviews is that group dynamics may shut down divergent views or prevent information about sensitive matters from being mentioned^12^. This study is limited to the experiences of self-recruited community midwives working in an urban area in Norway and the findings should therefore be interpreted with caution, especially in relation to rural settings.

## CONCLUSIONS

The midwives had both positive and negative experiences of an organizational change in early postpartum care services from hospital to home-based care in Norway. The study indicates a need for increased resources, training in new tasks and employees being more included when changes in midwifery practice are planned and implemented. The current study contributes knowledge of relevance to those planning to start home-based postpartum care which may contribute to improve quality of care, employees’ satisfaction and prevent burnout.

## Data Availability

The data supporting this research cannot be made available for privacy reasons.
